# Supplementary figures and images for: Maternal gut microbiota Bifidobacterium promotes placental morphogenesis, nutrient transport and fetal growth in mice
Source: Cell Mol Life Sci. 2022 Jun 28;79(7):386. doi: 10.1007/s00018-022-04379-y (PMC9236968; doi:10.1007/s00018-022-04379-y)

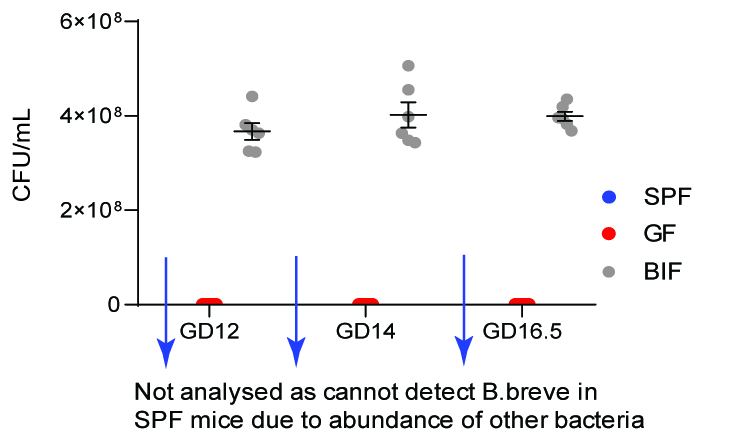

Supplement: Supplementary file 1 — Supplementary Figure 1. Colonization levels of B. breve determined in maternal faecal samples on gestational day (GD), 12 and 14. Analysis performed by two-ways ANOVA (****P<0.0001). Data displayed as mean ± SEM. Number of dams for GF and BIF groups are 5 and 6, respectively. Assessment was performed only on dams sacrificed at GD16.5 (TIF 740 KB) [file 18_2022_4379_MOESM1_ESM.tif]
