# Supplementary material for: Maternal gut microbiota Bifidobacterium promotes placental morphogenesis, nutrient transport and fetal growth in mice
Source: Cell Mol Life Sci. 2022 Jun 28;79(7):386. doi: 10.1007/s00018-022-04379-y (PMC9236968; doi:10.1007/s00018-022-04379-y)
Supplement: Supplementary file 2 — Supplementary Table 1. List of primers used for placental labyrinth zone qPCR (DOCX 15 KB) [file 18_2022_4379_MOESM2_ESM.docx]

| *Supplementary Table 1* | *Forward* | *Reverse* |
| --- | --- | --- |
| *Hprt* | *CAGGCCAGACTTTGTTGGAT* | *TTGCGCTCATCTTAGGCTTT* |
| *Ubc* | *GGAGTCGCCCGAGGTCA* | *AAAGATCTGCATCGTCTCTCTCAC* |
| *Vegf* | *GAAGCTACTGCCGTCCGATT* | *CTTCATCGTTACAGCAGCC* |
| *Akt* | *GCCGCCTGATCAAGTTCTCC* | *TTCAGATGATCCATGCGGGG* |
| *Mapk1* | *TGCTTTCTCTCCCGCACAAA* | *GGCCAGAGCCTGTTCAACTT* |
| *Mapk14* | *AGCTGTCGAGACCGTTTCAG* | *GATGGGTCACCAGGTACACG* |
| *Dlk1* | *GAAAGGACTGCCAGCACAAG* | *CACAGAAGTTGCCTGAGAAGC* |
| *Igf2P0* | *GAGGAAGCTCTGCTGTTTGG* | *CAAAGAGATGAGAAGCACCAAC* |
| *Slc38a1* | *CGGCGCCTTTCCCTTTATTTC* | *CCGTTAACTCGAGGCCACTT* |
| *Slc38a2* | *TTCTGATTGTGGTGATTTGCAAGAA* | *CAGGATGGGCACAGCATACA* |
| *Slc38a4* | *AAGGTAGAGGCGGGAAAGGG* | *AGGAACTTCTGACTTTCGGCA* |
| *Slc2a1* | *GCTTATGGGCTTCTCCAAACT* | *GGTGACACCTCTCCCACATAC* |
| *Slc2a3* | *GA TCGGCTCTTTCCAGTTTG* | *CAA TCA TGCCACCAACAGAG* |
| *Cd36* | *ATGGGCTGTGATCGGAACTG* | *GTCTTCCCAATAAGCATGTCTCC* |
| *Fatp1* | *GGCTCCTGGAGCAGGAACA* | *ACGGAAGTCCCAGAAACCAA* |
| *Fatp3* | *GAGAACTTGCCACCGTATGC* | *GGCCCCTATATCTTGGTCCA* |
| *Fatp4* | *GATTCTCCCTGTTGCTCCTGT* | *CCATTGAAGCAAACAGCAGG* |
| *Fatp6* | *AACCAAGTGGTGACATCTCTGC* | *TCCATAAAGTAAAGCGGGTCAG* |
